# Instability of Acylcarnitines in Stored Dried Blood Spots: The Impact on Retrospective Analysis of Biomarkers for Inborn Errors of Metabolism

**DOI:** 10.3390/ijns6040083

**Published:** 2020-11-02

**Authors:** Willemijn J. van Rijt, Peter C. J. I. Schielen, Yasemin Özer, Klaas Bijsterveld, Fjodor H. van der Sluijs, Terry G. J. Derks, M. Rebecca Heiner-Fokkema

**Affiliations:** 1 Section of Metabolic Diseases, Beatrix Children’s Hospital, University Medical Center Groningen, University of Groningen, 9700 RB Groningen, The Netherlands; w.j.van.rijt@umcg.nl (W.J.v.R.); t.g.j.derks@umcg.nl (T.G.J.D.); 2 Reference Laboratory for Neonatal Screening, Centre for Health Protection, National Institute for Public Health and the Environment, 3721 BA Bilthoven, The Netherlands; peter.schielen@rivm.nl; 3 Laboratory of Metabolic Diseases, Department of Laboratory Medicine, University Medical Center Groningen, University of Groningen, 9700 RB Groningen, The Netherlands; y.ozer@umcg.nl (Y.Ö.); k.bijsterveld@umcg.nl (K.B.); f.van.der.sluijs@umcg.nl (F.H.v.d.S.)

**Keywords:** acylcarnitine, dried blood spot, inborn error of metabolism, metabolite instability, metabolite stability, newborn blood spot screening, retrospective diagnosis, storage, tandem mass spectrometry

## Abstract

Stored dried blood spots (DBS) can provide valuable samples for the retrospective diagnosis of inborn errors of metabolism, and for validation studies for newborn blood spot screening programs. Acylcarnitine species are subject to degradation upon long-term storage at room temperature, but limited data are available on the stability in original samples and the impact on acylcarnitine ratios. We analysed complete acylcarnitine profiles by flow-injection tandem mass spectrometry in 598 anonymous DBS stored from 2013 to 2017, at +4 °C during the first year and thereafter at room temperature. The concentrations of C2-, C3-, C4-, C5-, C6-, C8-, C10:1-, C10-, C12:1-, C12-, C14:1-, C14-, C16:1-, C16-, C18:2-, C18:1-, C18-, C5OH+C4DC-, C18:1OH-, and C16DC-carnitine decreased significantly, whereas a positive trend was found for free carnitine. Only the C4/C8-, C8/C10-, C14:1/C10- and C14:1/C16-carnitine ratios appeared robust for the metabolite instability. The metabolite instability may provoke the wrong interpretation of test results in the case of retrospective studies and risk the inaccurate estimation of cut-off targets in validation studies when only stored control DBS are used. We recommend including control DBS in diagnostic, retrospective cohort studies, and, for validation studies, we recommend using fresh samples and repeatedly re-evaluating cut-off targets.

## 1. Introduction

The development of tandem mass spectrometry (MS/MS) enabled rapid determination of acylcarnitine and amino acid profiles [1,2]. This technique is commonly used in population newborn bloodspot screening (NBS) to screen for disorders of mitochondrial fatty acid oxidation, and organic acid and amino acid metabolism [3]. The primary process of NBS programs (e.g., screening panel, sampling window, analytical techniques, storage time of filter paper cards and storage conditions) varies worldwide, and even within countries [4,5]. In The Netherlands, 17 inborn errors of metabolism (IEM) are currently included in the NBS program [6]. The dried blood spots (DBS) on filter paper cards are generally stored for a maximum period of five years after laboratory analysis [6].

Many IEMs are associated with unexpected death in early childhood, resulting from energy deficiency or intoxication [7]. The often non-specific clinical presentations imply a risk of IEMs to remain unrecognized as cause of death. Stored DBS can provide a valuable sample for post-mortem investigations after unexpected death in early childhood [8,9,10]. This especially holds for countries, where only a limited selection of IEMs is included in the NBS program and/or diagnostic resources are scarce [4]. Moreover, stored DBS can be used for the evaluation of biomarkers of IEMs considered to be included in the NBS program [11].

A study we recently performed on the prevalence of IEMs in children who died in early childhood, using metabolite analysis in stored DBS, was complicated by the impact of long-term storage on the acylcarnitine profiles. While the stability of C3DC-, C5DC-carnitine and saturated acylcarnitine species has been analysed in stored, spiked DBS [12,13], the available data on acylcarnitine concentrations in stored, original DBS are limited and only concern the stability of C3DC-, C0-, C2-, C3, C4, and C5-carnitine [14,15,16]. Besides, the impact of metabolite stability on acylcarnitine ratios, which can be used for diagnostic differentiation and characterization of IEMs [3], has never been assessed. Hence, we systematically analysed the complete acylcarnitine profiles in original DBS stored from 2013 to 2017. Here, we report how metabolite instability can complicate the interpretation of retrospectively analysed acylcarnitine biomarkers for IEMs in stored DBS, and reflect on the potential risks of using control DBS stored at room temperature for validation studies for NBS programs.

## 2. Materials and Methods

The Medical Ethical Committee of the University Medical Center Groningen (Groningen, The Netherlands) confirmed that the Medical Research Involving Human Subjects Act did not apply and that official approval of this study by the Medical Ethical Committee was not required (METc code 2016/694). The Dutch NBS program is regulated by the National Institute for Public Health and the Environment (Bilthoven, The Netherlands) (RIVM in Dutch). Its Research Committee on Neonatal Screening (WONHS in Dutch) authorized the study. A waiver of consent was granted by both the METc and WONHS, since the study concerned anonymized samples.

In The Netherlands, neonatal blood, sampled from the heel, is spotted on filter paper cards between 72 and 168 h after birth and subsequently analysed in one of the five regional screening laboratories, according to national protocols [6]. All filter paper cards were stored at +4 °C for one year at the respective regional screening laboratory for quality assurance purposes. If authorized by the parents, the filter paper cards were stored at room temperature for another four years at the central archive of the RIVM reference laboratory, for quality assurance purposes and anonymized retrospective biomarker studies. There was no conditioning of humidity. The cards were stored in sealed bags (200–300 per bag) in cardboard boxes (20–30 bags per box). Once a year, in January of calendar year “X”, all filter paper cards of calendar year “X-6” are destroyed [6]. If the parents object to the use of residual anonymized blood samples for scientific research, the filter paper card is destroyed after one year.

This study is part of a larger research project on the prevalence of IEMs in children who died in early childhood. DBS included in the current study were from children with normal NBS results (i.e., control samples in the aforementioned study). In October 2018 (start of study), the available stored filter paper cards dated back to 2013. A total of 120 anonymized DBS on filter paper cards per complete storage year, i.e., 2013 to 2017, were retrieved from the storage facilities of the RIVM, resulting in five storage year cohorts (i.e., 2013, 2014, 2015, 2016 and 2017). Until analyses, the DBS were kept in sealed bags with freshly added silica sachets, stored in a refrigerator at the laboratory of Metabolic Diseases in Groningen, the Netherlands. The number of spots per year cohort, i.e., 120, permits the nonparametric determination of reference intervals [17,18]. Filter paper cards of children whose parents did not authorize the use for anonymous research purposes were excluded.

Flow-injection MS/MS analysis was used to quantitatively determine the acylcarnitine profiles in the DBS. Three 3.2 mm Ø discs were punched out of the DBS. Acylcarnitines were extracted by vortexing for 30 min at 600 rpm after the addition of 6 deuterium-labeled internal standards in methanol/MilliQ H_2_O (80/20 *v*/*v*). Supernatant was transferred to a vial and analyzed by flow-injection tandem-mass spectrometry (Sciex API4500, Framingham, MA, USA). Concentrations of C0 and the individual acylcarnitines were quantified by multiple reaction monitoring. C0 by an *m*/*z* transition of 162 ≥ 103 and acylcarnitines by selected precursor ions with *m*/*z* 85 as the common product ion, as described previously [19]. Data evaluation was performed with Analyst^®^ MD 1.6.2 Instrument Control and Data Processing Software (Sciex, Framingham, MA, USA). The measurements took place between November 2018 and July 2019. Quality control specimens were analysed parallel with the study samples to monitor the precision during the study period. Concentrations were expressed in micromole per liter, with two decimal places.

Data analysis was performed using SIMCA Software, version 15.0.2 (Umetrics, Umea, Sweden), Microsoft Excel with the Analyse-it add-in, version 4.81.6, and IBM SPSS Statistics for Windows, version 23 (IBM Corporation, Armonk, NY, USA). GraphPad Prism, version 5.0 (GraphPad Software, La Jolla, CA, USA) was used to create graphs. First, a principal component analysis was used to visualize and explore the complete dataset. Cases outside the 99% Hotelling’s T^2^ range were identified as strong outliers and excluded from further data analysis. Next, the distributions of the acylcarnitine concentrations and their molar ratios per storage year cohort were assessed. Jonckheere’s trend test was used to test for significant trends upon storage duration, a *p*-value of <0.05 was considered statistically significant. The test was not applied when >50% of the cases had concentrations at or below the detection limit (i.e., <0.01–0.05, depending on acylcarnitine species investigated), when >50% of the cases concerned zero or infinite values for molar ratios, or when a visual trend was absent. For the acylcarnitine species with a significant change in the concentration upon long-term storage, regression analysis was used to define the trend. For linear trends, the annual decay rates were estimated from the slope of the trend line equations. The four-year percent decays were calculated from the estimated decay rates and the median acylcarnitine concentrations in 2017. For other trend types, we calculated the four-year percent decays from the difference in the median concentrations in 2017 and 2013. Finally, we evaluated the possible consequences of metabolite instability for adequate interpretation of acylcarnitine biomarkers for IEMs upon long-term storage room temperature. To this aim, we investigated the potential impact on (1) retrospective MS/MS analysis in stored DBS for the detection of IEMs, and (2) validation studies in stored control DBS to determine cut-off targets for NBS programs.

## 3. Results

A total of 598 DBS were analysed; 119 DBS from storage years 2013 and 2014 and 120 DBS from storage years 2015–2017. The data of the low- and high-quality control samples per acylcarnitine species are depicted in Table S1. The mean inter-assay coefficient of variation was <20% for all acylcarnitine species, except for some acylcarnitines (i.e., unsaturated-, hydroxy-, dicarboxylic, and very long-chain acylcarnitine species) with concentrations close to the detection limit. Upon outlier detection via principal component analysis (*n* = 598, four components, UV scaling, cumulative R2X = 0.536; cumulative Q2 = 0.373), 20 cases were excluded from the dataset (i.e., 2014: *n* = 6/119; 2015: *n* = 6/120; 2016: *n* = 1/120; 2017: *n* = 7/120).

**Figure 1:**
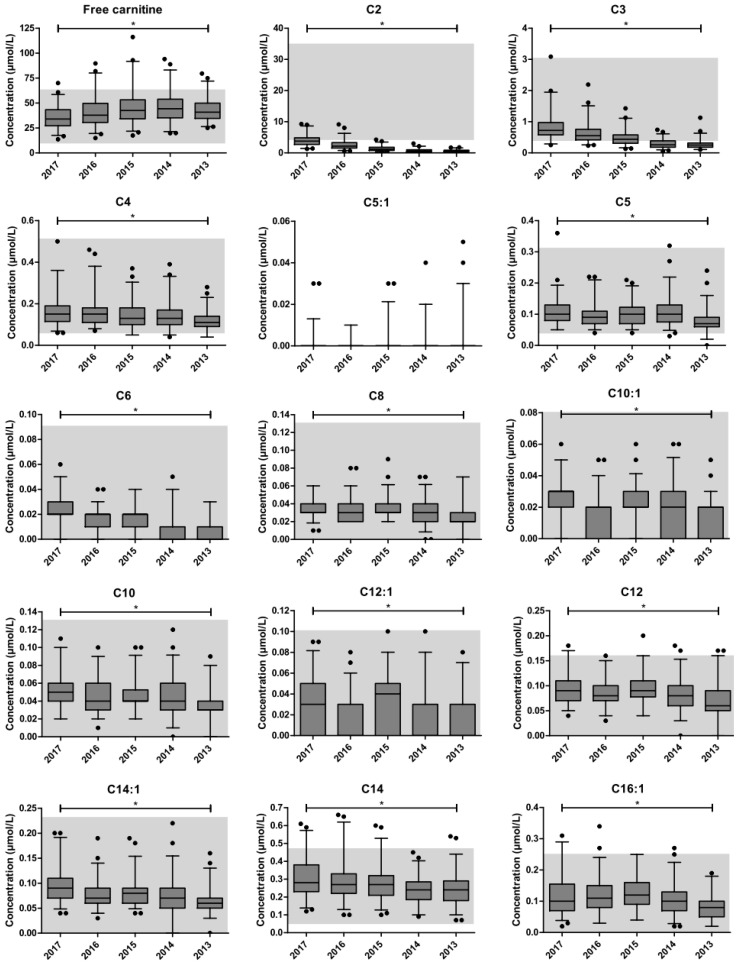

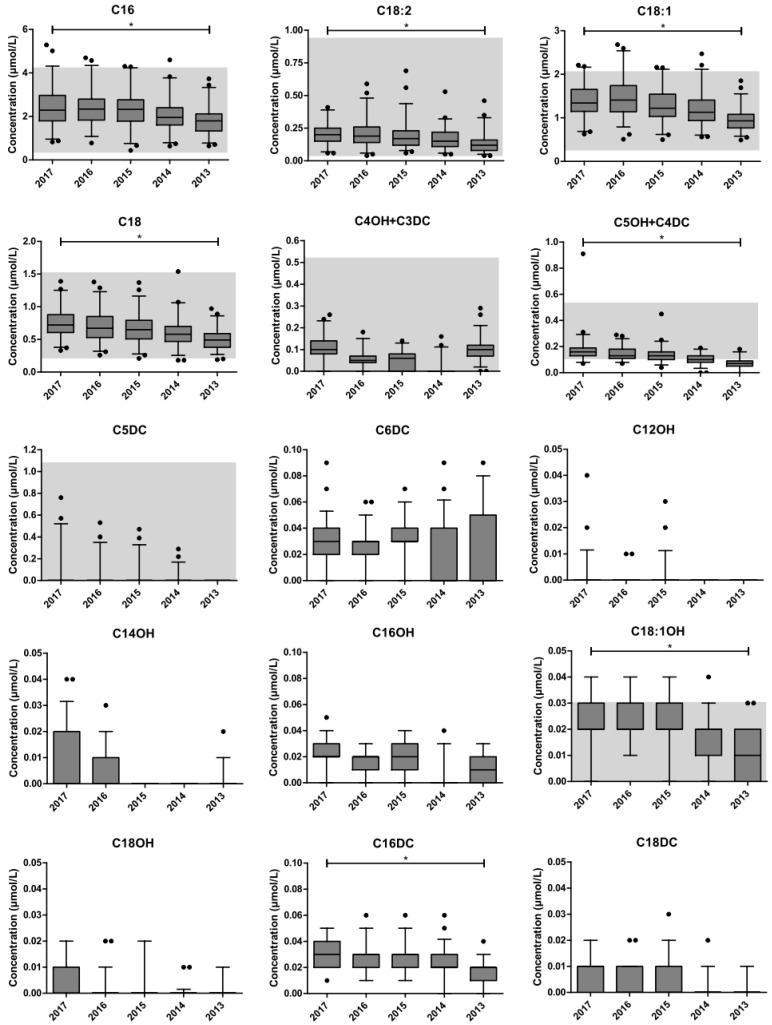

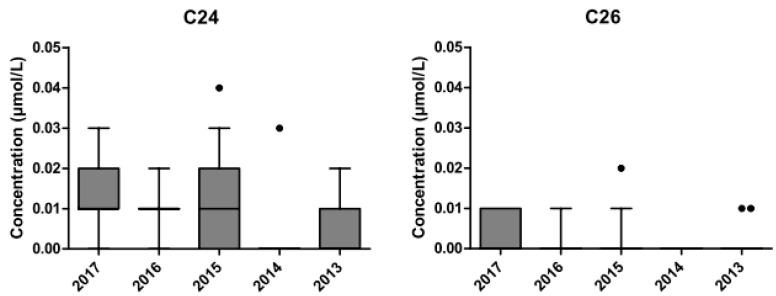
Changes in carnitine and acylcarnitine concentrations. The impact of metabolite instability upon long-term storage at room temperature. The boxplots represent the first quartile, median and third quartile. The whiskers extend to the 2.5th and 97.5th percentiles. Individual dots represent non-extreme outliers. Shaded areas represent the reference ranges as determined by our centre for the neonatal population. For C5:1-, C12OH-, C14OH-, C16OH-, and C18OH-, C6DC-, C16DC-, C18DC-, C24-, and C26-carnitine, the upper reference limit is below the detection limit (i.e., not detectable). * Statistically significant trend in the concentration upon storage duration, as determined by Jonckheere’s trend test. The following parameters were excluded from statistical analysis: C5:1-, C4OH + C3DC-, C5DC-, C6DC-, C12OH-, C14OH-, C16OH-, C18OH-, C18DC-, C24-, and C26-carnitine.

The changes in carnitine and acylcarnitine concentrations per storage year cohort are presented in Figure 1. Jonckheere’s trend test revealed a significant negative trend upon long-term storage for C2-, C3-, C4-, C5-, C6-, C8-, C10:1-, C10-, C12:1-, C12-, C14:1-, C14-, C16:1-, C16-, C18:2-, C18:1-, C18-, C5OH + C4DC-, C18:1OH-, and C16DC-carnitine concentrations. A positive trend was found for free carnitine. The total carnitine concentration (i.e., the sum of acylcarnitines plus free carnitine) did not change upon long-term storage.

Using regression analysis, the trend type for free carnitine was defined as polynomial, whereas, for C2- and C3-carnitine the trend appeared exponential. For the remaining acylcarnitine species, the trend was defined as linear. The estimated decays of the acylcarnitine species are presented in Table S2. The observed decreases in absolute concentrations between 2017 and 2013 were greatest for C2-, C3-, C16-, C18:1, C18-carnitine. The most substantial percentual drop in concentrations was found for C2-, C3-, C6-, C5OH + C4DC-, and C18:1OH-carnitine, with estimated four-year decays above 60%. The estimated four-year decay ranged between 22% and 34% for the remaining saturated acylcarnitines, and between 30% and 45% for the unsaturated acylcarnitines and C16DC-carnitine.

**Figure 2:**
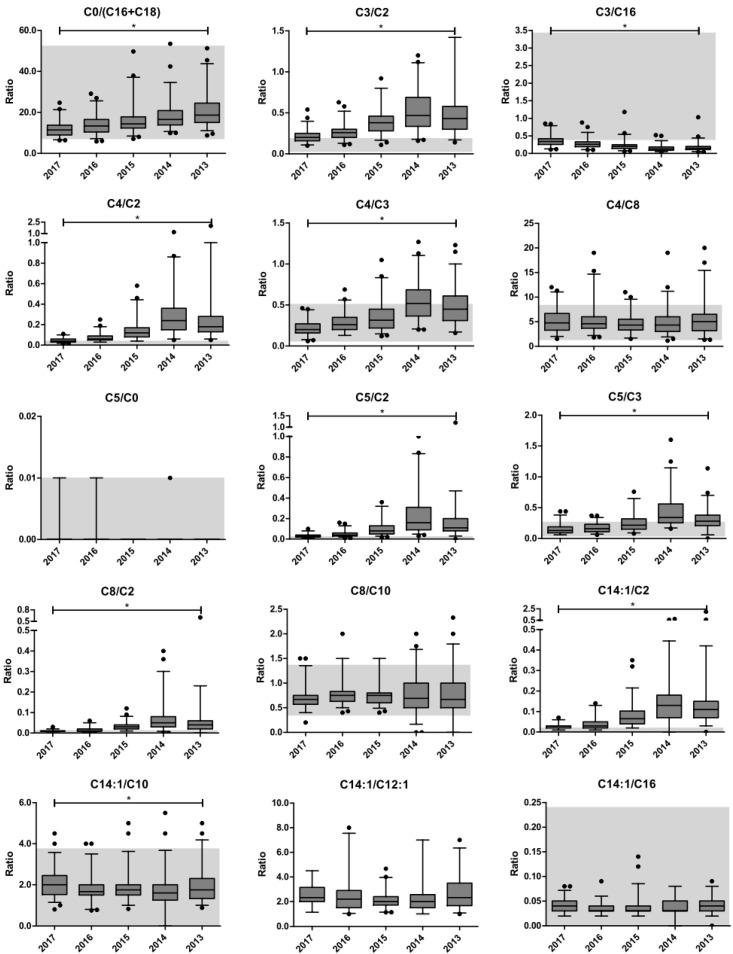

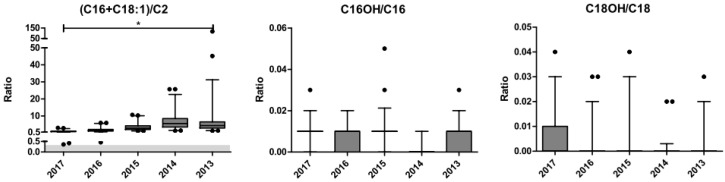
Changes in the molar ratios of carnitine and acylcarnitines. The impact of metabolite instability upon long-term storage at room temperature. The boxplots represent the first quartile, median and third quartile. The whiskers extend to the 2.5th and 97.5th percentiles. Individual dots represent non-extreme outliers. Shaded areas represent the reference ranges as determined by our centre for the neonatal population. For C14:1/C12:1, no reference range is available because of too many infinite values. For C16OH/C16 and C18OH/C18, the upper reference limit is 0.0. * Statistically significant trend in the molar ratio upon storage duration, as determined by Jonckheere’s trend test. The following ratios were excluded from statistical analysis: C5/C0-, C14:1/C12:1-, C16OH/C16-, and C18OH/C18-carnitine.

The impact of long-term storage on the molar ratios of acylcarnitine species is depicted in Figure 2. The C14:1/C12:1 ratio was excluded from statistical analysis because of infinite values in >50% of the data entries. Only the C4/C8-, C8/C10-, and C14:1/C16-carnitine ratios did not demonstrate a significant trend upon long-term storage, as determined by Jonckheere’s trend test. The C14:1/C10-carnitine ratio, though statistically significant, did not show a visual trend. The percentual decays of the individual acylcarnitines in these ratios were similar (i.e., the absolute difference in the estimated percent decays was <5%). Therefore, these ratios were assessed as robust with regard to the impact of metabolite instability upon long-term storage at room temperature.

The metabolite instability increases the likelihood of wrong interpretation of acylcarnitine biomarkers for IEMs in DBS stored at room temperature. Table 1 and Table 2 present the potential risks for the detection of IEMs upon retrospective MS/MS analysis, and for validation studies in stored control DBS to determine cut-off targets for NBS programs.

## 4. Discussion

Since the development of MS/MS analysis, several studies have reported on the instability of acylcarnitines upon long-term storage at room temperature. However, limited data are available on the decay in original samples, and the impact on acylcarnitine ratios and the associated risks of using common cut-off values for the interpretation of acylcarnitine profiles, have never been systematically assessed [12,13,14,15]. In this study, upon long-term storage at room temperature, we found a decrease in the measured concentrations of short-, medium-, and long-chain acylcarnitines of saturated and unsaturated fatty acids, while free carnitine increased. The total carnitine concentration remained similar throughout the years of storage. Likely, the acylcarnitine species are hydrolysed to free carnitine upon long-term storage at room temperature. The different decay rates of acylcarnitine species have a substantial impact on many molar ratios. In fact, only the C4/C8-, C8/10-, and C14:1/C16-carnitine ratios, and C14:1/C10-carnitine ratio, appeared robust for the metabolite instability.

In the case of retrospective, diagnostic analysis, metabolite instability may provoke false-positive and false-negative test results. Feasible identification of IEMs depends on the stability of the disease-related metabolite combined with its initial concentration and the significance of the elevation of the characteristic metabolite to the corresponding cut-off value. For many acylcarnitine biomarkers, at least the 1st to 10th percentile of the disorder range (or the 90th to 100th percentile in case of low cut-off targets) overlaps with reference intervals for the healthy population [3]. This interference, and thus the likelihood of overlooking an IEM, increases upon greater metabolite instability. Based on our results, the most adverse effects for reliable detection are expected for IEMs associated with accumulations of C3- and C5OH + C4DC-carnitine and free carnitine (e.g., propionic aciduria and methylmalonic aciduria; and 3-methylcrotonyl-CoA carboxylase deficiency, 3-hydroxy-3-methylglutaric aciduria, holocarboxylase synthetase deficiency, beta-ketothiolase deficiency; carnitine palmitoyltransferase I deficiency), and in case molar ratios are used as a primary screening test such as C14:1/C2, C0/(C16 + C18) and (C16/C18:1)/C2 (e.g., very long-chain acyl-CoA dehydrogenase deficiency; carnitine palmitoyltransferase I deficiency; and carnitine palmitoyltransferase II deficiency, carnitine-acylcarnitine translocase deficiency). Additionally, since >75% of the C5DC-carnitine measurements were below the detection limit, there appears to be a considerable impact on the retrospective diagnosis of glutaric aciduria type I. The detection of a carnitine uptake defect seems complicated by the substantial rise in free carnitine, but the sum of acylcarnitines (and free carnitine) may still enable the diagnosis [13]. In line with previous studies, the impact of metabolite instability appears relatively limited for the detectability of IEMs associated with saturated medium- and long-chain acylcarnitine accumulations, and when compared to the degradation of galactose-1-phosphate uridyltransferase and biotinidase activities [13,20]. In case of retrospective, diagnostic analysis, examination of the complete acylcarnitine profile may allow for an estimate of the metabolite instability. This would improve the interpretation of potential deviations and could reveal outliers that would otherwise not have been discovered. Especially in areas where the NBS panel is small and/or diagnostic resources are limited, this may be relevant. For diagnostic, retrospective cohort studies, we recommend including control DBS.

Caution is warranted when control DBS stored at room temperature are used for validation studies of NBS programs, because of the risk on incorrect estimation of reference ranges and cut-off targets. This particularly applies when, given their rarity, the storage conditions for samples of IEM cases for comparison analysis are different (e.g., frozen, different storage time). For validation studies, we therefore advocate using fresh control samples and comparing them to carefully stored samples of IEM cases and controls. Additionally, we emphasize the importance of repeated re-evaluation of a metabolite’s cut-off target after its introduction to the NBS panel.

Metabolite stability is affected by storage conditions. Increased temperature, humidity and sunlight exposure are known to influence sample integrity. This also accounts for other analytes used in NBS programs, such as amino acids, endocrine and enzyme markers [13,16,20,21,22]. Our results emphasize the importance of obtaining background information on a sample’s storage conditions. Whereas storage at room temperature over a two-year period results in substantial changes in the concentrations of several acylcarnitine and amino acid species, these changes are attenuated or not present upon storage at −20 or −80 °C [16]. To minimize metabolite instabilities, DBS storage at low humidity (<30%) and under frozen conditions (≤−20 °C) is recommended [13,16,20,22,23]. Moreover, during transport, the temperature and humidity should preferentially be minimized as much as possible [20]. Additionally, for optimal interpretation, the condition of the patient at the time of sample collection needs to be taken into account. For example, the subject’s age, metabolic stress and post-mortem sample obtainment can have a considerable impact on a metabolite profile [9,24].

Some study limitations deserve discussion. First, the initial acylcarnitine profiles are unknown due to the required anonymization procedures. This limited calculation of the true percent decays, which were instead estimated from the median concentrations in 2017. Second, there is a variation in the stability data because (1) the exact storage time of the individual DBS is unknown due to the required anonymization, and (2) the longitudinal timing of our analyses. There is evidence that suggests a more rapid degradation during the first months of storage at room temperature, which eventually slows down [12,13]. Therefore, the total decay might even be larger than the presented estimations, especially since the samples in our cohort were stored in a refrigerator without silica during the first year. Third, disparity in DBS quality and punch location are also important contributors to variation in analyte concentrations [25]. Fourth, it is unknown whether the efficiency of extraction of metabolites from filter paper cards changes upon the long-term storage of DBS. Possible effects on extractability do, however, not invalidate our findings, as the potential consequences coincide with the impact of metabolite instability. Finally, we had no access to NBS samples of diagnosed patients that were stored under the same conditions in order to validate the claimed risks presumably associated with the detection of IEMs in DBS upon long-term storage at room temperature.

In conclusion, acylcarnitine profiles in DBS stored at room temperature are subject to metabolite instability. In case of diagnostic, retrospective cohort studies, we recommend including control DBS. For validation studies, we recommend using fresh samples and to repeatedly re-evaluate cut-off targets.

## Figures and Tables

**Table 1:** Potential impact of metabolite instability on the interpretation of carnitine and acylcarnitine concentrations.

Parameter	Disorder	Retrospective Analysis of IEMs	Validation Studies for NBS Programs
		Risk Category	Potential Effect on Cutoff Target
C0 (low)	CUD	False-negative	Too high
C0 (high)	CPT-I	False-positive	Too high
C2 (low)	CUD, CPT-II	False-positive	Too low
C3 (low)	CUD	False-positive	Too low
C3 (high)	PROP, MUT, Cbl A-D	False-negative	Too low
C4	SCAD, EE, IBG, FIGLU ^a^, MADD	False-negative	Too low
C5	IVA, MADD, 2MBG, EE	False-negative	Too low
C6	MCAD, MADD	False-negative	Too low
C8	MCAD, MADD	False-negative	Too low
C10:1	MCAD	False-negative	Too low
C10	MADD, MCAD	False-negative	Too low
C12:1	MADD, VLCAD	False-negative	Too low
C12	MADD, CPT-II, CACT, VLCAD	False-negative	Too low
C14:1	VLCAD, MADD, LCHAD/TFP	False-negative	Too low
C14	MADD, CPT-II, VLCAD, CACT, LCHAD/TFP	False-negative	Too low
C16:1	VLCAD, LCHAD/TFP, CACT, CPT-II	False-negative	Too low
C16 (low)	CPT-I, CUD	False-positive	Too low
C16 (high)	CACT, CPT-II	False-negative	Too low
C18:2 (low)	CPT-I	False-positive	Too low
C18:2 (high)	CPT-II, CACT	False-negative	Too low
C18:1 (low)	CPT-I, CUD	False-positive	Too low
C18:1 (high)	CPT-II, CACT	False-negative	Too low
C18 (low)	CPT-I, CUD	False-positive	Too low
C18 (high)	CPT-II, CACT	False-negative	Too low
C5OH + C4DC	3MCC, HMG, MCD, 3MGA, BTD, BKT, 2M3HBA	False-negative	Too low
C18:1OH	LCHAD/TFP	False-negative	Too low
C16DC	PBD	False-negative	Too low

The potential consequences associated with (1) the interpretation of retrospectively analysed carnitine and acylcarnitine concentrations in DBS upon long-term storage at room temperature for the detection of IEMs, and (2) using control DBS stored at room temperature for validation studies on cut-off targets for NBS programs. ^a^ FIGLU is only associated with a high C4-carnitine when butylation is applied. Abbreviations (in alphabetical order): 2M3HBA, 2-methyl-3-hydroxybutyric aciduria (i.e., alpha-methylacetoacetic aciduria; online mendelian inheritance in man (OMIM) #203750); 2MBG, 2-short/branched chain acyl-CoA dehydrogenase deficiency (# 610006); 3MCC, 3-methylcrotonyl-CoA carboxylase deficiency (#210200, #210210); 3MGA, 3-methylglutaconic aciduria (#250950, #302060); BKT, beta-ketothiolase deficiency (#203750); BTD, biotinidase deficiency (#253260); CACT, carnitine-acylcarnitine translocase deficiency (#212138); Cbl, cobalamin deficiency (complementation group); CPT-I, carnitine palmitoyltransferase I deficiency (#255120); CPT-II, carnitine palmitoyltransferase II deficiency (#255110); CUD, carnitine uptake defect (#212140); EE, ethylmalonic encephalopathy (#602473); FIGLU, formiminoglutamic aciduria (#229100); IBG, isobutyryl-CoA dehydrogenase deficiency (#611283); IVA, isovaleryl-CoA dehydrogenase deficiency (#243500); HMG, 3-hydroxy-3-methylglutaric aciduria (#246450); LCHAD, long-chain L-3-Hydroxy dehydrogenase deficiency (#609016); MADD, multiple acyl-CoA dehydrogenase deficiency (#231680); MCAD, medium-chain acyl-CoA dehydrogenase deficiency (#607008); MCD, holocarboxylase synthetase deficiency (#253270); MUT, methylmalonic aciduria (#251000, 251100, 251110); PBD, peroxisome biogenesis disorder (complementation group); PROP, propionic aciduria (#606054); SCAD, short-chain acyl-CoA dehydrogenase deficiency (#201470); TFP, trifunctional protein deficiency (#609015); VLCAD, very long-chain acyl-CoA dehydrogenase deficiency (#201475).

**Table 2:** Potential impact of metabolite instability on the interpretation of carnitine and acylcarnitine ratios.

Molar Ratio	Disorder ^a^	Retrospective Analysis of IEMs	Validation Studies for NBS Programs
		Risk Category	Potential Effect on Cutoff Target
C0/(C16 + C18) (low)	CPT-II, CACT	False-negative	Too high
C0/(C16 + C18) (high)	CPT-I	False-positive	Too high
C3/C2	PROP, MUT, Cbl A-D, MCD	False-positive	Too high
C3/C16	PROP, MUT, Cbl A-D, CPT-I, MCD	False-negative	Too low
C4/C2	SCAD, MADD, IBG, EE, FIGLU ^b^	False-positive	Too high
C4/C3 (low)	MCD, Cbl A-D, PROP	False-negative	Too high
C4/C3 (high)	EE, IBG, FIGLU ^b^, MADD, SCAD	False-positive	Too high
C4/C8	IBG, SCAD, EE, FIGLU ^b^	None, similar percent decay of the involved acylcarnitine species	
C5/C2	IVA, MADD, 2MBG, EE	False-positive	Too high
C5/C3 (low)	MCD, MUT, Cbl A-B, PROP	False-negative	Too high
C5/C3 (high)	IVA, MADD, EE, 2MBG	False-positive	Too high
C8/C2	MCAD, MADD	False-positive	Too high
C8/C10	MCAD	None, similar percent decay of the involved acylcarnitine species	
C14:1/C2	VLCAD, MADD, LCHAD/TFP	False-positive	Too high
C14:1/C10	VLCAD	Appears negligible, negative statistical trend, but no visual trend and similar percent decay of the involved acylcarnitine species	
C14:1/C16	VLCAD, MADD, LCHAD/TFP	None, similar percent decay of the involved acylcarnitine species	
(C16 + C18:1)/C2 (low)	CPT-I	False-negative	Too high
(C16 + C18:1)/C2 (high)	CPT-II, CACT	False-positive	Too high

The potential consequences associated with (1) the interpretation of retrospectively analysed carnitine and acylcarnitine ratios in DBS upon long-term storage at room temperature for the detection of IEMs, and (2) using control DBS stored at room temperature for validation studies on cut-off targets for NBS programs. Shaded rows represent the ratios that appear robust for the metabolite instability. The percent decays were defined as similar if the absolute difference in the estimated percent decays was <5%. The C14:1/C12:1 ratio was excluded from analysis because of infinite values in >50% of the data entries. ^a^ See caption of Table 1 for the defined abbreviations. ^b^ FIGLU is only associated with a high C4-carnitine when butylation is applied.

## Supplementary Materials

The following are available online at https://www.mdpi.com/2409-515X/6/4/83/s1. Table S1. The inter-assay precision per acylcarnitine species. Table S2. The estimated decay rates of free carnitine and acylcarnitine species upon long-term storage.

## Abbreviations

DBS	Dried blood spot
IEM	Inborn errors of metabolism
MS/MS	Tandem mass spectrometry
NBS	Newborn bloodspot screening
