# Supplementary material for: Instability of Acylcarnitines in Stored Dried Blood Spots: The Impact on Retrospective Analysis of Biomarkers for Inborn Errors of Metabolism
Source: Int J Neonatal Screen. 2020 Nov 2;6(4):83. doi: 10.3390/ijns6040083 (PMC7712882; doi:10.3390/ijns6040083)
Supplement: Supplementary file 1 [file IJNS-06-00083-s001.pdf]

Table S1. The inter-assay precision per acylcarnitine species. Calculated from quality control samples (n = 30 in ten experiments). The coefficients of variation (CV) were calculated as the ratio of the standard deviation to the mean. For C5:1-, C12OH-, C14OH-, C18OH-, C18DC-, C24- and C26-carnitine, concentrations were below the limit of detection (shown as not detectable: ND). Acylcarnitines in the low or high QC samples with concentrations below the limit of quantification are in *italic*.

Parameter	Low QC			High QC		
	Average (µmol/L)	SD	CV (%)	Average (µmol/L)	SD	CV (%)
**Total**	48.75	3.38	6.9	69.02	4.68	6.8
**C0**	25.50	2.09	8.2	29.84	2.38	8.0
**C2**	16.74	1.06	6.3	21.49	1.36	6.3
**C3**	1.94	0.13	6.6	6.17	0.36	5.9
**C4**	0.18	0.02	10.6	0.40	0.04	9.6
**C5:1**	ND			*0.01*	*0.01*	*161.3*
**C5**	0.08	0.01	15.4	0.24	0.03	11.8
**C6**	0.02	0.00	18.5	0.28	0.03	10.4
**C8**	*0.04*	*0.01*	*22.6*	1.94	0.17	8.6
**C10:1**	*0.02*	*0.01*	*38.4*	0.36	0.04	10.8
**C10**	0.05	0.01	18.9	0.48	0.04	8.1
**C12:1**	*0.01*	*0.01*	*191.8*	0.09	0.02	17.4
**C12**	*0.05*	*0.01*	*27.8*	0.29	0.03	10.8
**C14:1**	*0.05*	*0.01*	*23.1*	0.37	0.04	9.7
**C14**	0.15	0.02	14.2	0.30	0.02	7.7
**C16:1**	0.08	0.01	14.2	0.18	0.02	10.9
**C16**	0.96	0.08	7.9	1.14	0.08	7.1
**C18:2**	0.31	0.03	10.2	0.42	0.04	9.0
**C18:1**	1.26	0.11	8.9	1.73	0.14	8.1
**C18**	0.49	0.05	9.8	1.00	0.09	8.8
**C4OH+C3DC**	*0.10*	*0.06*	*61.6*	*0.20*	*0.14*	*70.4*
**C5OH+C4DC**	0.32	0.03	10.4	0.51	0.07	13.3
**C5DC**	*0.05*	*0.12*	*231.7*	0.92	0.16	17.8
**C6DC**	*0.01*	*0.01*	*141.2*	0.08	0.01	17.4
**C12OH**	ND			ND		
**C14OH**	ND			ND		
**C16OH**	*0.01*	*0.01*	*120.5*	*0.01*	*0.01*	*141.1*
**C18:1OH**	*0.01*	*0.01*	*136.6*	*0.02*	*0.01*	*43.9*
**C18OH**	ND			ND		
**C16DC**	*0.02*	*0.01*	*51.4*	*0.03*	*0.01*	*28.8*
**C18DC**	ND			ND		
**C24**	ND			*0.01*	*0.01*	*63.4*
**C26**	ND			*0.01*	*0.01*	*95.0*

Table S2. The estimated decay rates of free carnitine and acylcarnitine species upon long-term storage. Estimated decay rates and percent decays for carnitine and the acylcarnitine concentrations with a significant trend upon storage duration, as determined by Jonckheere's trend test. For free carnitine, the trend type was defined as polynomial trend, whereas for C2- and C3-carnitine the trend appeared exponential. For the remaining acylcarnitine species, the trend type was defined as linear. Abbreviation: ND, not determined.

Parameter	Estimated annual decay rate (µmol/L (95% CI))	Estimated annual percent decay from 2017 (% (95% CI))	Estimated percent decay 2017 – 2013 (%(95% CI))
**C0**	ND	ND	-20.0%
**C2**	ND	ND	84.4%
**C3**	ND	ND	65.3%
**C4**	0.010 (0.006 – 0.014)	6.7% (4.3 – 9.1 %)	26.7% (17.1 – 36.3%)
**C5**	0.005 (0.003 – 0.008)	5.5% (3.2 – 7.7%)	21.8% (12.7 – 30.9%)
**C6**	0.004 (0.004 – 0.005)	20.8% (17.8 – 23.9%)	83.4% (71.1 – 95.6%)
**C8**	0.002 (0.001 – 0.003)	6.0% (3.5 – 8.5%)	24.0% (13.9 – 34.1%)
**C10:1**	0.002 (0.002 – 0.003)	7.6% (5.1 – 10.1%)	30.4% (20.4 – 40.4%)
**C10**	0.004 (0.002 – 0.005)	7.1% (5.0 – 9.2%)	28.4% (19.9 – 36.8%)
**C12:1**	0.003 (0.002 – 0.004)	10.4% (5.9 – 14.9%)	41.5% (23.4 – 59.7%)
**C12**	0.006 (0.004 – 0.007)	6.3% (4.5 – 8.1%)	25.2% (17.8 – 32.5%)
**C14:1**	0.007 (0.005 – 0.009)	7.6% (5.7 – 9.5%)	30.4% (22.8 – 37.9%)
**C14**	0.016 (0.011 – 0.022)	5.8% (3.8 – 7.7%)	23.1% (15.2 – 30.9%)
**C16:1**	0.009 (0.006 – 0.012)	9.4% (6.5 – 12.4%)	37.7% (26.0 – 49.5%)
**C16**	0.158 (0.114 – 0.202)	6.9% (5.0 – 8.8%)	27.6% (20.0 – 35.2%)
**C18:2**	0.019 (0.014 – 0.024)	9.5% (11.8 – 7.1%)	38.0% (47.4 – 28.6%)
**C18:1**	0.116 (0.095 – 0.137)	8.6% (7.1 – 10.2%)	34.6% (28.3 – 40.9%)
**C18**	0.062 (0.050 – 0.073)	8.5% (6.9 – 10.2%)	34.2% (27.6 – 40.7%)
**C5OH+C4DC**	0.024 (0.021 – 0.028)	15.2% (13.3 – 17.2)	60.9% (53.1 – 68.8%)
**C18:1OH**	0.003 (0.003 – 0.004)	15.4% (18.1 – 12.6%)	61.4% (72.3 – 50.6%)
**C16DC**	0.003 (0.003 – 0.004)	11.3% (9.3 – 13.4%)	45.3% (37.0 – 53.5%)
